# The immune system’s role in attention deficit hyperactivity disorder development

**DOI:** 10.1007/s00787-026-02973-0

**Published:** 2026-03-04

**Authors:** Leandro Lemos Ferreira, Laiana Azevedo Quagliato

**Affiliations:** Institute of Psychiatry, Federal University of Rio de Janeiro, Avenida Venceslau Brás, Botafogo, Rio de Janeiro, 22290-140 Brazil

**Keywords:** Immune, Adhd, Pathophysiological, Inflammatory

## Abstract

Attention deficit hyperactivity disorder (ADHD) is one of the most common disorders in minors. Although its symptoms are well-described, the mechanisms regarding physiopathology are not yet fully known. Most studies in the literature are focused on the genetic and neurochemical aspects that differ in this population in comparison to healthy subjects. However, some data suggest that ADHD might be linked to alterations in the immune system as well, which have not been largely investigated so far. The aim of this cross-sectional study is to investigate baseline levels of inflammatory and oxidative stress markers and DNA damage in a cohort of children with ADHD and a healthy control group. Serum interleukin-1 beta (IL-1β), thiobarbituric acid reactive substances (TBARS) and 8-hydroxy-2-deoxyguanosine (8-OHdG) levels were measured. The results revealed significantly higher levels of IL-1β (t = 4.099; df = 80; *p* < 0.001), TBARS (t = 13.629; df = 85; *p* < 0.001) and 8-OHdG (t = 10.382; df = 85; *p* < 0.001) in ADHD patients than in healthy controls. This study suggests that the immune system is likely involved in the pathophysiological development of ADHD.

**Supplementary information:**

The online version contains supplementary material available at 10.1007/s00787-026-02973-0.

## Introduction

Attention deficit hyperactivity disorder (ADHD) is a condition that initiates in childhood and it is marked by attention, impulsivity and agitation problems. Studies in the field so far have indicated that ADHD is probably associated to neural circuitry deficiencies, genetic polymorphisms and brain alterations that lead to the described symptoms [1, 2].

One of the best described and studied pathophysiological cause for impairment in patients with ADHD is the malfunctioning roles of dopamine and norepinephrine in the regulation of corticostriatal circuits, which impacts in executive function development [1]. Genome-wide studies have also found that several *loci* in the genome of a large sample of participants with ADHD had variants of DNA that increased the risk of the disorder, one of them being FOXP2 gene, also known to be involved in the risk of language deficits [2]. Besides, neuroimaging studies have reported that patients with ADHD probably have smaller brain volumes comparing to healthy controls [3].

All of these findings indicate that the pathophysiology of ADHD involves several underlying mechanisms not yet discovered, which could also involve inflammatory alterations and impairments in the immune system. This suggestion is reinforced by two literature reviews with a large population size that found patients with ADHD were more likely to have autoimmune disorders compared to healthy subjects [4, 5]. Therefore, more studies are needed to understand how the immune system could or not be associated with this disorder.

Oxidative and inflammatory pathways have been increasingly implicated in the neurobiology of attention-deficit/hyperactivity disorder (ADHD), although, to date, little is known about which inflammatory and immune parameters could represent potential biomarkers. The markers examined in this study were selected based on their relevance to mechanisms underlying attention, impulse control, and neurodevelopment. Lipid peroxidation indices known as thiobarbituric acid reactive substances (TBARS), which have malondialdehyde (MDA) as its main product, have already been described to be elevated in autism spectrum disorder population [6] and ADHD [7, 8]; its mechanism reflects oxidative injury to polyunsaturated fatty acid–rich neuronal membranes that may impair dopaminergic and noradrenergic neurotransmission in fronto-striatal circuits critical for attention control [8]. Similarly, oxidative DNA damage measured by 8-hydroxy-2′-deoxyguanosine (8-OHdG) has been reported in children and adults with ADHD and indicates disruption of cellular redox homeostasis during critical periods of brain maturation [9, 10]. In parallel, pro-inflammatory cytokines such as interleukin-1β (IL-1β) are often altered in ADHD and play central roles in microglial activation, synaptic pruning, and modulation of excitatory–inhibitory balance [11–14]. Together, these findings provide a biologically plausible framework for examining TBARS, 8-OHdG, and IL-1β as candidate biomarkers of pathophysiological processes potentially contributing to ADHD-related symptom expression.

Therefore, the primary aim of the present study was to investigate baseline levels of these inflammatory and oxidative stress markers and DNA damage in a well-characterized, unmedicated, somatically healthy cohort of children with ADHD, controlling for relevant confounders. Based on previous research pertaining to this, our a priori hypotheses were that the specific oxidative stress marker TBARS; the concentration of 8-OHdG, representing DNA damage; and the inflammatory marker IL-1β would be elevated in unmedicated patients with ADHD compared to controls. The second aim is to evaluate the relationship between IL-1β, TBARS, and 8-OHdG metabolites.

## Methods

### Study design and participants

This was a cross-sectional study with a matched sample of drug-naïve healthy children. For this study, drug-naïve patients with ADHD (*n* = 41) were selected. Participants aged 6 years to 17 years and 11 months were eligible to enter the study. Data were collected between April 2023 and December 2024. Individuals were included in the ADHD group if they (1) had been diagnosed with ADHD without any comorbidity, and (2) parents reported no lifetime psychiatric medication use and no present or former psychotherapy. Drug-naïve status was defined as having never received psychotropic medicine or psychotherapy in one’s life, as evidenced by universal electronic medical records and corroborated in the patient’s interview. A healthy control group (*n* = 46) was also recruited. The healthy control group was required to have no present or previous mental health issues, evaluated by the Child Behavior Checklist (CBCL) [15] and through an interview with psychiatrists. Potential participants were also excluded if they had uncontrolled cardiovascular, endocrinological, hematological, hepatic, renal, or neurological diseases; autoimmune conditions; chronic infections (i.e., HIV, hepatitis B or C); a history of liver abnormalities; or evidence of infection of their respective treatments, such as steroids, antiretroviral therapy, anti-inflammatory therapy, or chemotherapy, within one month of screening. These illnesses and therapies may lead to a bias in the interpretation of the study’s results. Participants were matched based on their sex and age.

### Clinical assessments

The diagnosis of ADHD was determined by a structured clinical interview based on the Diagnostic and Statistical Manual of Mental Disorders, Fifth Edition (DSM-5) [16], administered by a trained psychiatrist or psychologist, and independently confirmed by a research psychiatrist. The Swanson, Nolan, and Pelham Rating Scale (SNAP-IV) [17] was administered to all patients to obtain measures of general psychopathology and to evaluate total symptom severity. All assessments were made by psychiatrists trained in the administration and scoring of the assessments. The height and weight of all participants were recorded for body mass index (BMI) calculation. Household income mean was also assessed. The procedures were explained, and written informed consent was obtained from parents’ participants prior to participation in the study, which was approved by the research ethics committee of the Federal University of Rio de Janeiro. This study was performed in accordance with the ethical standards of the Declaration of Helsinki (Ethics approval ID: CAAE 13970819.4.0000.5263).

### IL-1β, TBARS and 8-OHdG levels

Blood samples were collected in the morning (8 a.m. ± 1 h) using EDTA tubes via catheter after subjects rested for at least 30 min. Fasting was not requested. The samples were promptly centrifuged (1000 x g for 10 min), and the serum was extracted and preserved at − 80 °C until batch analysis. The Immulite System (Diagnostic Products Corporation) was employed to determine IL-1β concentrations. This method utilizes a solid-phase two-site chemiluminescent enzyme immunometric assay. A polystyrene bead, serving as the solid phase, is coated with either a specific monoclonal antibody or an anti-ligand. Patient serum was mixed with an alkaline-phosphatase-conjugated monoclonal antibody or a ligand-labeled antibody, depending on the procedure, and incubated for 30–60 min at 37 °C. Unbound conjugate was eliminated through a triple centrifugal wash, followed by a 10-minute incubation of the test unit with a chemiluminescent substrate (adamantyl dioxetane phosphate ester). The substrate undergoes hydrolysis in the presence of alkaline phosphatase, producing an unstable intermediate that emits light. The luminometer measures the photon output of the bound complex, which is proportional to the sample’s cytokine concentration. For each cytokine calibration, the manufacturer created a master curve using material calibrated against National Institute for Biological Standards and Control standards.

Serum 8-OHdG levels were assessed using an enzyme immunoassay protocol with a DNA Damage Competitive Elisa Kit (Thermo Fisher Scientific, USA), following the manufacturer’s guidelines. This protocol initiates the binding reaction by adding a peroxidase-labeled mouse monoclonal antibody to 8-hydroxy-2’-deoxyguanosine to each well. After a 1-hour incubation and washing, substrate was introduced. The substrate interacts with the peroxidase-labeled antibody that has bound to the conjugate. Following a brief incubation, the reaction was halted, and the resulting color intensity was measured using a microtiter plate reader at 450 nm. Lipid peroxidation was quantified using a TBARS Assay kit (Cayman Chemical, Ann Arbor, MI, USA) in accordance with the manufacturer’s instructions. This method involves the reaction of MDA with thiobarbituric acid (TBA) under high temperature (90–100 °C) and acidic conditions, forming the MDA-TBA adduct. The presence of this adduct was determined fluorometrically at an excitation wavelength of 530 nm and an emission wavelength of 550 nm. All measurements were conducted using a Synergy 2 multimode microplate reader and Gen5 Software (BioTek, Winooski, VT, USA). Each assay was performed in duplicate, with intra- and interassay coefficients of variation below 7.5% for all analytes.

### Statistical analysis

Statistical analysis was performed using the Statistical Package for the Social Sciences (SPSS) version 27.0. The normality of the distribution of the variables was tested using the Kolmogorov–Smirnov test. Independent- sample t tests were used for parametric variables, and Mann–Whitney U tests were used for nonparametric variables. Statistical significance was set at *p* < 0.05. Multiple linear regression using stepwise methods were used to assess the relationship of IL-1β levels and TBARS, and 8-OHdG levels with several potentially confounding variables related to sociodemographic, clinical, and physical characteristics (sex, age, ethnicity, household income, BMI, and SNAP-IV) as independent variables. Recent stress-related events that might affect inflammatory profiles (trauma, bullying, or other acute stressors) prior to participant inclusion were screened, and no participants had experienced such exposures recently. We used Bonferroni correction to control for multiple comparisons (a total of 7 variables were introduced; thus, the p-value threshold was set at 0.05/7 = 0.007). Furthermore, multivariate linear regression models using stepwise methods were performed to evaluate if IL-1β and TBARS could predict 8-OHdG levels in children with ADHD.

Before data collection, we conducted an a priori power analysis using G*Power 3.1 to estimate the minimum sample size required for detecting between-group differences. Assuming a medium effect size (Cohen’s d = 0.50), α = 0.05, and desired statistical power of 0.80, the analysis indicated that a minimum of 64 participants (32 per group) would be sufficient for detecting clinically meaningful effects commonly reported in the ADHD literature.

Our final sample exceeded this target, comprising 41 drug-naive individuals with an ADHD diagnosis and 46 healthy controls (total *N* = 87). The groups were successfully matched, with no significant demographic differences. Therefore, the achieved sample provided adequate power to test our hypotheses with precision and robustness.

## Results

### Relationship of IL-1β, TBARS and 8-OHdG levels in patients with ADHD and controls and their association with demographic factors

The sample was composed of 41 drug-naive individuals with an ADHD diagnosis and 46 healthy controls. The sample was successfully matched, and there were no significant differences between groups regarding demographic characteristics, as shown in Table 1. There were no significant associations of IL-1β, TBARS and 8-OHdG levels with any confounding variables, such as sex, age, ethnicity, BMI and SNAP-IV scores.

**Table 1:** Demographic characteristics and IL-1β, TBARS and 8-OHdG levels of study participants

Variable (mean and standard deviation)	ADHD (*n* = 41)	Controls (*n* = 46)	Difference	*p*-value	95% CI^f^	Cohen’s d
Age in years	9.5 (3.5)	9.0 (2.8)	ns*			
Sex (male; female)	30; 11	34; 12	ns			
Ethnicity	34 Brown 5 white 2 black	36 Brown 6 white 4 black				
BMI^a^	16.3 (2.9)	15.4 (2.5)	ns			
Household income	R$ 1.5087,00 (2.500,00)	R$ 1.890,00 (1.560,00)	ns			
SNAP-IV^b^	Hyperactivity/impulsivity 19.9 (6.01) Inattention 21.3 (4.6) Oppositional/defiant disorder 10.8 (4.13)					
IL-1β ^c^ (pg/ml)	3.6854 (4.09628)	0.9780 (1.05213)	t = 4.099; df = 80	*p* < 0.001	1.03–4.02	0.905
TBARS^d^ (µmol/l)	86433.3171 (18371.27221)	11638.5867 (30548.84993)	t = 13.629; df = 85	*p* < 0.001	(9.38–13.8)	2.23
8-OHdG^e^ (pg/ml)	1186.4634 (749.06732)	25.2574 (116.31461)	t = 10.382; df = 85	*p* < 0.001	(6.38–8.57)	2.92

T-tests were performed to compare IL-1β, TBARS and 8-OHdG levels in ADHD patients compared to controls. Abbreviations: *ns non-significant, *t* t test, *df* difference, *a* body mass index, *b* Swanson, Nolan, and Pelham Rating Scale, *c* interleukin-1 beta, *d* thiobarbituric acid reactive substances, *e* 8-hydroxydeoxyguanosine, *f* 95% confidence interval

The analysis of serum IL-1β, TBARS and 8-OHdG levels revealed significantly higher levels of IL-1β (t = 4.099; df = 80; *p* < 0.001), TBARS (t = 13.629; df = 85; *p* < 0.001) and 8-OHdG (t = 10.382; df = 85; *p* < 0.001) in patients with ADHD than in healthy controls (Table 1).

### Relationship between IL-1β, TBARS and 8-OHdG levels in patients with ADHD

The multiple regression model showed that there are no associations between the value of the biomarkers in ADHD group (refer to Table 1 in the Supplementary Material, which presents the absence of coefficients).

## Discussion

Our results showed that IL-1β, TBARS and 8-OHdG levels in the ADHD group were higher than in the control group, which suggests that patients with ADHD have a higher DNA damage, oxidative stress and inflammatory markers in comparison to healthy subjects.

**Fig. 1:**
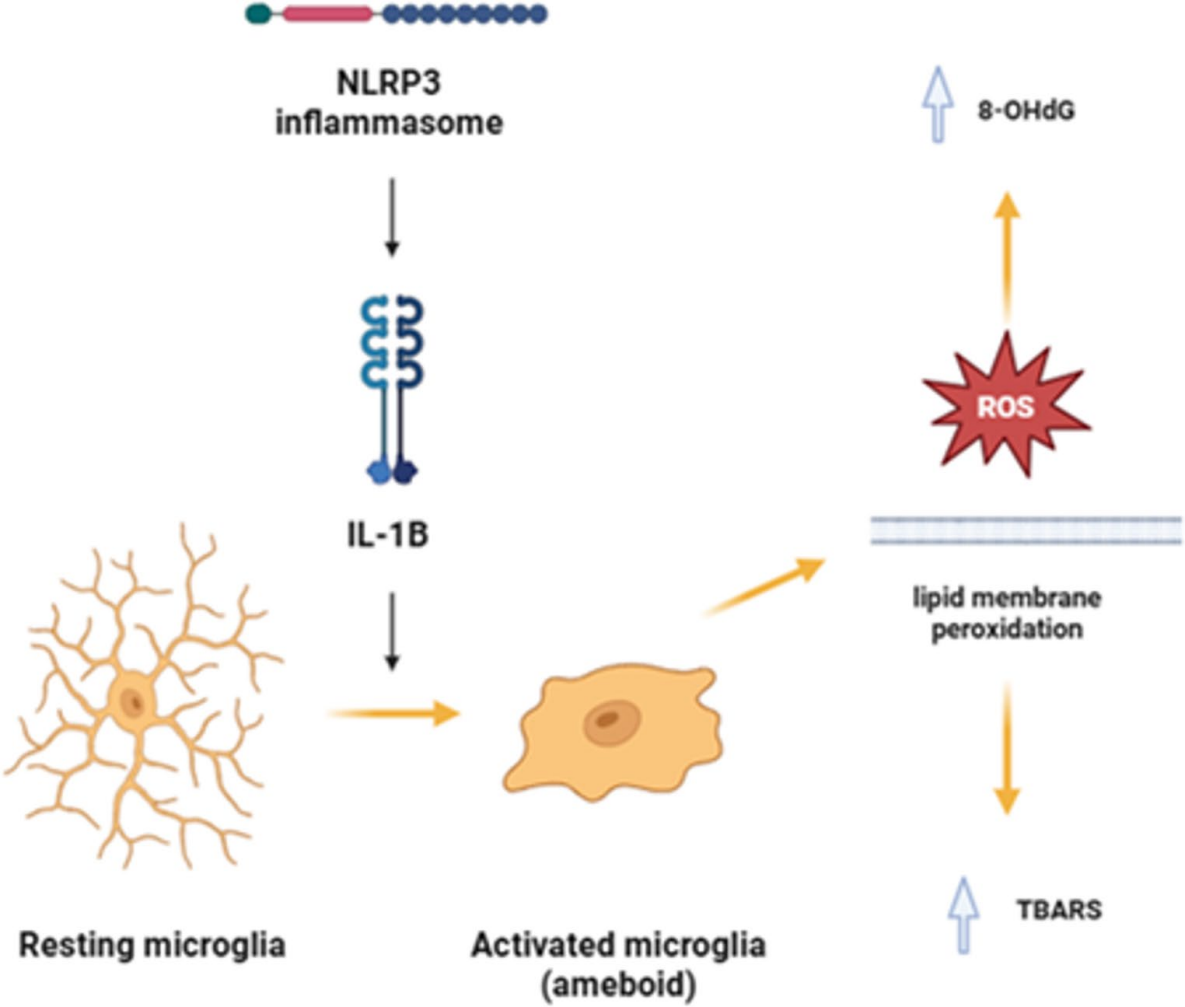
NRLP3 inflammasome theoretically activates the resting microglia via IL-1β. The activated microglia produces reactive oxygen species (ROS) markers that react with the lipidic membrane, causing lipid peroxidation, leading to higher levels of TBARS and DNA damage markers, as 8-OHdG

The present findings should be interpreted within the broader framework of neuroinflammatory mechanisms involving microglial activation, which are shown in a conceptual model represented in Fig. 1 below. A substantial body of evidence supports the central role of microglia as key mediators of inflammatory responses in the central nervous system. In this context, as Fig. 1 shows, activation of microglia is related to the expression of the NLRP3 inflammasome, leading to caspase-1–dependent cleavage of pro-IL-1β into its mature form. This pathway represents a well-characterized mechanism by which innate immune signaling is amplified in the brain and has been consistently demonstrated across experimental models of neuroinflammation [18–20]. IL-1β is a potent pro-inflammatory cytokine whose actions extend beyond microglia to influence astrocytes, neurons, and endothelial cells. Although IL-1β signaling can contribute to the maintenance and amplification of inflammatory states [21], it is important to note that microglial activation is regulated by multiple converging signals, and the extent to which IL-1β acts in a sustained autocrine feedback loop likely varies according to the inflammatory context [22].

Activated microglia have also been shown to produce reactive oxygen species (ROS), thereby contributing to oxidative stress within the neural microenvironment. Oxidative stress, in turn, has been associated with lipid peroxidation and oxidative DNA damage in brain tissue [23]. In the present conceptual model, markers such as thiobarbituric acid reactive substances (TBARS) and 8-hydroxy-2′-deoxyguanosine (8-OHdG) are interpreted as indicators of increased oxidative damage rather than as direct or microglia-specific outcomes of inflammasome activation. These measures reflect cumulative tissue-level oxidative processes and may arise from multiple cellular sources, including neurons and other glial populations. Therefore, while the involvement of microglial activation and NLRP3 inflammasome signaling in neuroinflammatory processes is strongly supported by existing literature, the downstream association with lipid peroxidation and DNA damage should be considered indirect and context-dependent [24, 25].

The levels of 8-OHdG are considered a marker for assessing DNA damage and few studies to date have investigated the relationship between its levels and children with ADHD. 8-OHdG reflects oxidative modification of guanine bases in DNA, which can interfere with neuronal proliferation, chronic oxidative stress and synaptic plasticity [26, 27]; there’s some evidence its modified levels could also be associated to impulsivity measures, increased oxidative stress and lower antioxidant enzymes [28], all of those representing mechanisms capable of interference with neurodevelopment. One previous study has observed a trend for higher urinary 8-OHdG levels [11] in this population than in controls. Higher blood 8-OHdG levels were also observed in a cohort of adults with ADHD as compared to the subjects in the healthy control group [10]. However, this pattern was not observed in another study conducted a few years earlier, in which the levels of the oxidant parameters measured, including 8-OHdG, were statistically lower in children with ADHD compared to controls [29]. The discrepant findings regarding 8-OHdG levels in ADHD likely reflect the dynamic and heterogeneous nature of oxidative stress across development, symptom profiles, and methodological approaches. Variations in compensatory antioxidant responses, DNA repair efficiency, ADHD subtype composition, biological matrices, and assay sensitivity may all contribute to the observed inconsistencies, rather than indicating absence of oxidative involvement.

Growing evidence indicates that oxidative stress might play a significant role in the pathophysiology of neurodevelopmental disorders, as a recent study investigating its role in children and adolescents with ADHD showed that the oxidative stress index (OSI) was significantly higher in the ADHD group, including IL-1β levels [30]. Two other studies evaluating the oxidative metabolism in children and adolescents also found that not only OSI, but total oxidant status (TOS) as well were both higher in children and adolescents with ADHD [31, 32]. Regarding lipid-peroxidation marker thiobarbituric acid reactive substances (TBARS), commonly used as a proxy for malondialdehyde (MDA), they have also been repeatedly implicated in this process [6]. Studies in children with autism spectrum disorder consistently report elevated TBARS or MDA levels, often accompanied by reductions in antioxidant defenses (e.g., glutathione, superoxide dismutase), suggesting increased vulnerability of polyunsaturated fatty acid–rich neuronal membranes to oxidative damage [33, 34]. Similarly, some findings have been described in attention-deficit/hyperactivity disorder, where elevated TBARS/MDA has been interpreted as reflecting heightened lipid peroxidation during critical periods of brain maturation [8, 35], though not universal, since some authors have found no difference or even lower levels instead, leading reviewers to characterise the evidence as suggestive but inconsistent [29, 36].

A major contributor for inconsistency is the limited specificity of the TBARS assay, which captures a range of aldehydic products beyond malondialdehyde and is highly sensitive to pre-analytical factors such as sample handling, storage, and fasting status. Moreover, heterogeneity in study design and populations—including differences in age ranges, developmental stage, ADHD subtype, comorbidities, and medication exposure—complicates cross-study comparability. Variability in biological matrices (e.g., serum, plasma, erythrocytes) and analytical protocols further adds to between-study differences. Collectively, these methodological and sample-related factors are likely to underlie the mixed TBARS findings reported in ADHD rather than reflecting true biological contradiction. Despite methodological and result variability limits the specificity and independence of TBARS as a diagnostic biomarker, its recurrent elevation across neurodevelopmental conditions underscores the broader contribution of oxidative stress to disrupted neurodevelopmental trajectories, positioning TBARS as a plausible supportive marker of oxidative imbalance.

Among the scarce other studies searching for inflammatory markers in infant and adolescent patients with ADHD, one study focusing on interleukins found that adolescents with ADHD showed increased levels of IL-1β, IL-16, TNF-a, M1 profile, proinflammatory profile and the proinflammatory/anti-inflammatory cytokine ratio, indicating a predominant activation of the inflammatory response system in adolescents with ADHD in both sexes in comparison to the control group [12]. Similarly, two systematic reviews searching for the role of inflammation in patients with ADHD identified that the co-occurrence of ADHD with inflammatory disorders has been demonstrated in a large sample of subjects [37, 38]. In fact, in the systematic review and meta-analysis covering the publication period until 30th September 2021 searching for serum levels of C-reactive protein and cytokines in subjects with ADHD and healthy controls, the levels of IL-6 were significantly higher in studies of participants up to the age of 18 years (k = 10, g = 0.70, 95%CI: 0.10–1.30, *p* = 0.023) and after including those above the age of 18 years as well (k = 10, g = 0.71, 95%CI: 0.12–1.31, *p* = 0.019) [38].

Emerging evidence suggests that aberrant IL-1β signalling may contribute to the pathophysiology of ADHD by disrupting fundamental neurodevelopmental processes. Although heterogeneity across studies must be considered — particularly as recent meta-analyses in youths report largely unaltered circulating IL-1β levels [13, 14, 38] — this variability may reflect differences in age, clinical phenotype, medication exposure, comorbid inflammatory conditions, and methodological factors such as assay sensitivity, biological matrix, and timing of cytokine measurement. In this context, the elevated IL-1β levels observed in the present study may indicate immune dysregulation in a biologically or clinically distinct subgroup of individuals with ADHD. Importantly, increased IL-1β has high neurodevelopmental relevance, as it can impair neuronal proliferation and differentiation, alter axonal and dendritic outgrowth, and interfere with synaptogenesis and circuit formation during critical developmental windows [39]. Moreover, IL-1β modulates microglial activation and synaptic pruning, potentially shifting excitatory–inhibitory balance within fronto-striatal and prefrontal networks involved in attention and impulse control [13, 37]. Finally, IL-1β is thought to influence dopaminergic and noradrenergic neurotransmission through neuroinflammatory pathways, providing a mechanistic link between immune alterations and the catecholaminergic deficits characteristic of ADHD [13].

It is interesting to note that epidemiological data suggest that atopic eczema in infancy might increase the risk for ADHD later in life [40]. Children with atopic eczema are susceptible to increased levels of proinflammatory cytokines and mediators, such as IL-1β, which a growing number of studies suggest that may induce in turn the secondary activation of other cytokines in the brain [21, 41]. This inflammatory cascade amplifies neuroinflammatory processes and can interfere with critical neurobiological functions, including synaptic plasticity, memory, and behavior, as demonstrated in several experimental models [42, 43]. Such interference in critical regions of the brain as those in the prefrontal cortex, known to subserve executive cognitive functions and believed to be linked to various cognitive disturbances including ADHD symptomatology, may render the child vulnerable to develop the disorder later in life.

Taken together, all these findings provide a biologically plausible framework in which higher levels of TBARS, 8-OHdG and IL-1β in children may increase inflammatory brain processes such as chronic oxidative stress, altered neuronal functioning and dysregulated neurotransmission pathways mediated by dopamine and norepinephrine, thus contributing to neurodevelopmental changes that might culminate in the ocurrence of ADHD.

Some limitations are present in this study. Methodological and biological heterogeneity likely contribute substantially to the inconsistent findings observed across studies examining TBARS/MDA, IL-1β and 8-OHdG in neurodevelopmental disorders. Differences in assay platforms (e.g., colorimetric vs. HPLC-based TBARS; ELISA vs. LC-MS/MS for 8-OHdG; multiplex vs. single-analyte cytokine assays), detection limits, sample type (serum, plasma, erythrocytes, urine), and pre-analytical conditions such as fasting state, collection timing, storage, and haemolysis introduce considerable measurement variability. For instance, since our study did not require fasting, future studies should consider implementing a fasting requirement when assessing biomarkers sensitive to metabolic fluctuations. At the same time, clinical heterogeneity—including broad age ranges, differing diagnostic subtypes, comorbidities, medication use, BMI and metabolic status, diet, sleep problems, psychosocial stress, and environmental exposures—affects oxidative and inflammatory profiles in ways that may amplify or obscure group differences. Together, these laboratory and clinical sources of variability create substantial noise across studies, complicating direct comparisons and likely explaining why some cohorts show clear elevations in these biomarkers while others report modest or null effects.

As a cross-sectional work, this study design also limits our interpretation since it’s not possible to establish causality once patients in both groups were not followed for a period of time to reassess if the alterations found in their serum had any change in the future or whether they were already present long before this study’s assessment. Dietary patterns were not directly assessed in the present data, which may have influenced inflammatory markers levels as well. The role of these markers in ADHD, in turn, is also limited so far regarding the disorder’s pathophysiological profile. Given that ADHD has distinct subtypes and is highly heterogeneous in its presentation, it is possible that different inflammatory profiles exist depending on the specific clinical phenotype. It is also worth noting that since our results only demonstrate variations in peripheral levels of the investigated markers, it still remains unclear if they actually represent brain inflammatory or oxidative processes. Besides, given the small size, further studies are needed in order to identify if our findings are corroborated by similar studies in the field.

## Supplementary information

Below is the link to the electronic supplementary material.

ESM  (DOCX 14.9 KB)

## Data Availability

Available on request.
