# Supplementary material for: The immune system’s role in attention deficit hyperactivity disorder development
Source: Eur Child Adolesc Psychiatry. 2026 Mar 4;35(6):1891–8. doi: 10.1007/s00787-026-02973-0 (PMC13337609; doi:10.1007/s00787-026-02973-0)
Supplement: Supplementary file 1 — (DOCX 14.9 KB) [file 787_2026_2973_MOESM1_ESM.docx]

**Appendix 1: Supplementary Table 1**

Supplementary Table 1. Stepwise Linear Regression with 8-OHdG as Dependent Variable (TBARS and IL-1β tested as predictors)

| Model | Predictor | B (unstd.) | SE B | β (std.) | | t | | p | 95% CI for B | Entered into model? |
| --- | --- | --- | --- | --- | --- | --- | --- | --- | --- | --- |
| Stepwise (entry p < .05) | TBARS | — | — | — | — | | — | | — | **No** (did not meet entry criterion; p > .05) |
| Stepwise (entry p < .05) | IL-1β | — | — | — | — | | — | | — | **No** (did not meet entry criterion; p > .05) |
| Final model summary | Intercept-only model | — | — | — | — | | — | | — | **No predictors entered** |
